# Does acute cannabidiol (CBD) use impair performance? A meta-analysis and comparison with placebo and delta-9-tetrahydrocannabinol (THC)

**DOI:** 10.1038/s41386-024-01847-w

**Published:** 2024-03-25

**Authors:** Lindsay A. Lo, April L. Christiansen, Justin C. Strickland, Carly A. Pistawka, Lauren Eadie, Ryan Vandrey, Caroline A. MacCallum

**Affiliations:** 1 Dalla Lana School of Public Health, University of Toronto, Toronto, ON Canada; 2 School of Medicine, Queen’s University, Kingston, ON Canada; 3 Faculty of Medicine, University of Toronto, Toronto, ON Canada; 4 Johns Hopkins University School of Medicine, Baltimore, MD USA; 5 Faculty of Science, University of British Columbia, Vancouver, BC Canada; 6 Department of Medicine, University of British Columbia, Vancouver, BC Canada

**Keywords:** Cognitive neuroscience, Medical research

## Abstract

Cannabidiol (CBD) is widely used and believed to be non-intoxicating, lacking acute performance effects (e.g., non-impairing). However, a synthesis of data has not evaluated this. This meta-analysis synthesized data from controlled human laboratory studies that evaluated if acute CBD use impairs performance. Performance on objective and subjective measures of cognitive and psychomotor function were used as markers for potential performance changes and impairment. Studies were identified through systematic database searches. Adult clinical trials measuring acute CBD effects (within 0–8 h of administration) were included. The primary outcome was the peak mean difference in performance measures between CBD and placebo. A secondary analysis utilizing delta-9-tetrahydrocannabinol (Δ9-THC) as a positive control for comparison to CBD was completed. Pooled Hedges’ *g* estimates were calculated using robust variance estimation (RVE) meta-regression. The omnibus RVE meta-analysis indicated a statistically significant, but small effect size (Hedge’s g < 0.2) for impaired performance following acute CBD consumption compared to placebo (*N* = 16 trials, Hedges’ *g* = 0.122, 95% CI: 0.023–0.221, *p* = 0.019). Measure type was a significant moderator with larger mean differences between CBD and placebo when subjective measures, specifically self-reported sedation, were used versus objective performance tasks (Hedges’ *g*_Subjective_ = 0.288 versus Hedges’ *g*_Objective_ = 0.048). Δ9-THC had a significantly greater magnitude of impairment compared to CBD (*N* = 8, Hedges’ *g* = 0.416, 95% CI: 0.017–0.816, *p* = 0.043). In summary, acute CBD consumption was associated with a small increase in subjective ratings of sedation, but no difference from placebo was observed across multiple domains of objectively assessed cognitive or psychomotor performance. These findings suggest that acute CBD alone is unlikely to significantly impair daily functioning or workplace performance.

## Introduction

The global increase in legal access to cannabis and cannabinoid-based products for medical and non-medical purposes has been paralleled by their widespread promotion and use. In particular, North America has the highest rate of cannabis use in the world, where some of the first jurisdictions to legalize cannabis exist [1–3]. In recent years, North America has been reported to have the highest prevalence of past-year cannabis use compared to other sub-regions globally at 14.5% [4]. Of the emerging cannabinoids available, cannabidiol (CBD), a principal cannabinoid presumed to be non-euphoric and non-intoxicating, has the highest prevalence of past-year use [5]. Past-year CBD use was reported to be 26.1% in the United States and 16.2% in Canada [6]. Common reported reasons for use include medical indications such as the management of pain, anxiety, and depression [6].

This surge in consumption and access to cannabis and cannabinoid products has sparked concerns regarding their careful use during ‘safety-sensitive’ work or activities (e.g., operating motor vehicles or machinery) [7, 8]. To date, a majority of research and public policy has focused on identifying and mitigating cannabis impairment risk related to delta-9-tetrahydrocannabinol (Δ9-THC), the main psychoactive cannabinoid known to produce acute deficits in cognitive performance and driving ability [9, 10]. In contrast, little attention has been given to CBD due to the general belief that it is non-impairing. Although the available evidence has pointed to a lack of cognitive, psychomotor, or subjective effects with oral and vapourized CBD even at high or supratherapeutic doses [11–13], there has yet to be a comprehensive, systematic review of the literature to synthesize data on the performance effects of acute CBD exposure, or evaluation of potential moderating factors that may impact sensitivity to performance effects.

This lack of clarity surrounding the effects of CBD on daily functioning presents several concerns. A primary concern is the potential public health consequences for traffic safety if people using CBD are operating motor vehicles under the assumption that it is non-impairing. It is equally as important to consider the implications of CBD-related impairment on workplace health, safety, and policy. At present, many workplaces err on the side of caution and treat cannabis as a single entity, subjecting CBD to the same restrictions as Δ9-THC [14, 15]. These restrictions are particularly relevant to people who use CBD for medical purposes to help manage symptoms or a condition, such as chronic pain, epilepsy, or anxiety when Δ9-THC cannot be used safely [16, 17]. In this context, CBD use may afford individuals the ability to engage in daily activities and workplace duties which they may otherwise be unable to do. Hence, efforts to clarify the risk of CBD-associated impairment are greatly needed to inform public health legislation, as well as workplace policy and practice.

The current meta-analysis synthesized and critically evaluated available evidence from human laboratory studies assessing the potential for CBD to impair cognitive and psychomotor performance. These effects were compared to a placebo control group and a positive control of Δ9-THC. Moderators were also evaluated to determine individual difference and product-specific factors that may alter the magnitude of effect.

## Methods

### Search strategy

This review was registered on PROSPERO (The International Prospective Register of Systematic reviews) (CRD42021247522) and reported in accordance with PRISMA guidelines (eTable 1) [18]. A systematic search in AMED, EMBASE, CENTRAL, PsychINFO, CINAHL, Clinicaltrials.gov, Medline, MedRxiv, and Web of Science was completed on June 22nd, 2022 and updated again on January 4th, 2023. Literature searches using the keywords associated with cannabidiol or CBD paired with cogniti*, or impair*, or domain-specific keywords (e.g., memory) were independently conducted by two reviewers. As an example, studies were identified by the following expression: (cogniti* OR driving OR coordinat* OR processing speed OR reaction time OR executive function OR memory OR “task performance and analysis” OR attention OR learn* OR task switching OR intoxic* OR motor OR impair* OR perform*) AND (cannabidiol* OR CBD OR Epidiolex OR Epidyolex). The star symbol (*) was used to capture derivatives of search terms (by suffixation) and enclosed quotation marks were used to capture exact phrases. See eTable 2 for full search strategies.

### Eligibility criteria and study selection

For inclusion, studies had to meet the following criteria: (1) involve adult participants; (2) placebo-controlled experimental design; (3) report route of cannabinoid administration and dose schedule; (4) measures of self-report, researcher observation, or objective neurocognitive or psychomotor assessments within 0–8 h of CBD administration. CBD administration was defined as administration of any form of CBD either in isolation or with a THC content of <1%. Self-reported/subjective measures of neurocognitive performance were restricted to those with specific constructs (e.g., alert, sedation). Subjective ratings of drug high or intoxication were excluded due to lack of specificity. Only full-length, English-language original research articles were accepted. Studies were excluded if: (1) performance test(s) were not administered within 8 h of CBD administration; (2) either the dose of CBD administered, or the length of time between CBD administration and the performance test(s), was not reported and could not be estimated (e.g., in regard to dose, reporting the number of ‘puffs’ smoked from a cannabis cigarette was not considered adequate to estimate dose); (3) there was no confirmation of a ≥24-h abstinence period for intoxicating substances (e.g., cannabis, alcohol, other recreational drugs) before performance assessments. See eTable 3 for PICOS statement. Three authors (LL, LE, and AC) assessed study eligibility and quality blinded, and resolved any disagreement by consensus. Authors LL, CP, and LE screened titles and abstracts. Authors LL and AC assessed full texts for eligibility and quality.

### Data extraction and outcome measures

Studies were required to have measured driving performance, a discrete cognitive skill (e.g., information processing), and/or subjective cognitive or psychomotor function. These performance outcomes were used as markers of potential impairment. Each cognitive test used in the included studies was categorized into a performance domain (Table 1). Categorizations of measures by cognitive function/domain were based on previous meta-analyses, to allow for greater comparability across the literature [9, 19]. All outcome measures of neurocognitive function or psychomotor performance on objective or subjective assessments were extracted for each domain (e.g., reaction time, accuracy of responses, mean score etc.). Additional variables were extracted including study participant characteristics, dose, product type, method of administration, concomitant drugs, comorbidities, cannabis experience, and type of performance assessment. The primary outcome was the peak mean difference in acute performance measures between CBD and placebo, as quantified by Hedges’ *g*. The secondary outcome was the peak mean difference in acute performance measures between CBD and Δ9-THC. Eligible effect estimates for the peak mean difference in studies with multiple time points were constrained to 0–120 minutes post-inhaled cannabis and 30–240 min post-oral cannabis consumption given the pharmacokinetics of each route of administration [13, 20, 21]. See eMethods for further details.

**Table 1:** Cognitive performance tests and associated domains.

Test	Neurocognitive performance domains
Digit Symbol Substitution Task (DSST)	Information processing^a^ Psychomotor function Attention
Divided Attention Task (DAT)	Divided Attention^a^ Psychomotor function
On road or simulated driving	Driving performance^a^
Driving Under the Influence of Drugs (DRUID^Ⓡ^) app	Psychomotor impairment Psychomotor vigilance Divided attention Spatial perception Visuomotor coordination
Tower of London (TOL)	Decision-making Executive function - cognitive flexibility Working memory^a^
Paced Auditory Serial Addition Task (PASAT)	Information processing Working memory^a^
Visual Analog Mood Scale (VAMS)	(1) Subjective feelings of sedation: alert vs. drowsy, attentive vs. dreamy^a^ (2) Subjective feelings of Cognitive impairment: quick-witted vs. mentally slow, proficient vs. incompetent, energetic vs. lethargic, clear headed vs. muzzy, gregarious vs. withdrawn, well-coordinated vs. clumsy, strong vs. feeble
Visual Analog Scale (VAS)	(1) Subjective feelings of Sedation: alert vs. drowsy, attentive vs. dreamy^a^ (2) Subjective feelings of internal perception: internal feelings that do not correspond with reality (3) Subjective feelings of external perception: misperception of external stimuli or changes in the awareness of the environment
Digit Span Task (DST)	Working memory^a^
Hopkin’s Verbal Learning Test (HVLT)	Episodic memory^a^
Spatial N-back Task	Working memory^a^ - spatial working memory (0-back=attention, 1-back=working memory, 2-back=working memory)
Prose recall (immediate and delayed)	Episodic memory^a^
Cancellation test	Attention Concentration Information processing^a^
Differential aptitude test	Attention Concentration Information processing^a^
Time production task	Perception^a^ - internal perceptual function
Finger tap test	Undirected upper limb motor speed
Delayed discounting	Executive function^a^ - impulsivity
Psychomotor vigilance test (PVT)	Sustained attention^a^
Trail-making test A/B	Working memory^a^ Information processing^a^ Task sequencing and shifting
Letter-Number Sequencing task	Working memory^a^
D-2 Test of Attention	Sustained attention^a^ Concentration
Verbal/semantic/language fluency task	Fluency^a^
Visual Oddball Detection Paradigm (VODT)	Perception^a^ - sensory discrimination
Verbal Paired Associative Learning (VPAL)	Episodic memory^a^
Go/No-Go	Executive function^a^ - conflict control

^a^Primary domain for analysis based on previous literature.

### Effect size computation

Hedges’ *g* effect estimates were calculated from the standardized mean difference (SMD) between matched intervention groups (CBD, Δ9-THC, placebo). Hedges’ *g* was used to provide a more unbiased estimate for small sample sizes [22]. All effect sizes were recorded such that positive Hedges’ *g* values indicated a greater magnitude of impaired performance. Effect sizes were interpreted using the convention (*g* = 0.2 [small], 0.5 [medium], 0.8 [large]) [23]. In order to compute Hedges’ *g*, Cohen’s *d* was first computed using the formula [24]:

$$d=\frac{{\bar{Y}}_{{diff}}}{{S}_{{within}}}=\frac{{\bar{Y}}_{1}-{\bar{Y}}_{2}}{{S}_{{within}}}$$

The standard deviation within groups was imputed from the standard deviation of the difference using the formula:

$${S}_{{within}}=\frac{{S}_{{diff}}}{\sqrt{2\left(1-r\right)}}$$

where *r* is the correlation between pairs of observations. If *r* was not reported or unable to be calculated from raw data, the standard 0.5 assumption was used. Cohen’s *d* was then converted to Hedges’ *g* using the formula:

$$g=J\times d$$

The J conversion factor was computed using the formula:

$$J=1-\frac{3}{4{df}-1}$$

### Meta-analytic methods

Omnibus effect estimates and moderation analyses were conducted using a robust variance estimation (RVE) meta-regression approach. The RVE approach allows for the incorporation of dependent effect size measurements (e.g., multiple effect sizes from crossover studies or studies with multiple outcome measures for the same participants) without violating independence assumptions by using robust standard errors based on heteroskedasticity-robust estimates and clustered methods (see refs. [25, 26] for details). This analysis utilized a modified RVE method for small-sample size adjustments [26]. Moderator analyses were carried out for the primary outcome of peak mean difference between CBD and placebo in a series of one-covariate analyses. Model outputs were not interpreted if the degrees of freedom were <4, as recommended by Hedges et al., 2010.

Sensitivity analyses were conducted using a standard random-effects meta-regression approach. In studies with multiple effects, effect estimates were averaged to produce a single effect. Traditional publication bias measures (e.g., Egger’s plot for funnel asymmetry) were conducted on the average effect size model as they have not yet been widely validated for RVE models.

All analyses were carried out in *R* using the robumeta [27] and Metafor packages [28].

### Risk of bias and quality assessment

All studies were assessed for risk of bias using the revised Cochrane Risk of Bias (RoB) tool [29]. The RoB 2.0 comprises five domains, including the randomization process, deviation from intended interventions, missing data, measurement of the outcome, selective outcome reporting, and “other sources of bias”. Two independent assessors (LL and AC) performed the risk of bias assessments, with any disagreement resolved by consensus. A decision around the interpretability of the available evidence was made by categorizing studies by the research question and rating them based on their quality.

## Results

### Study characteristics

**Fig. 1:**
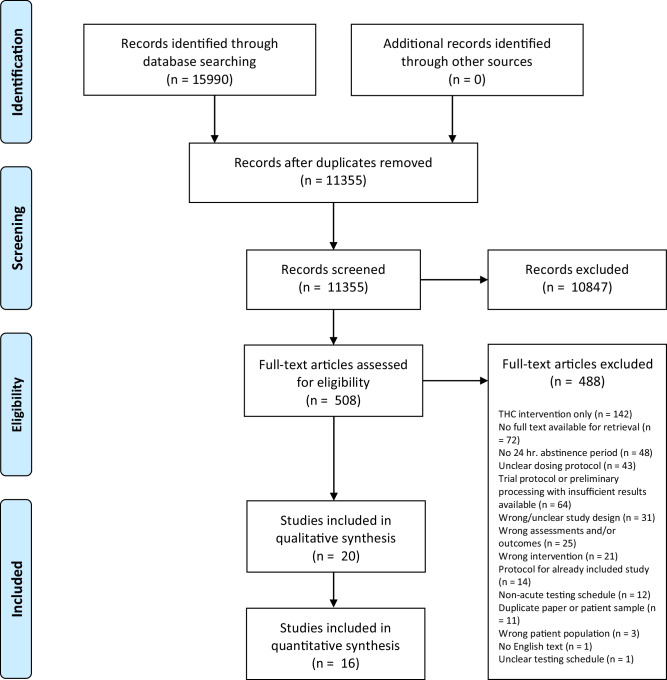
PRISMA flow diagram.

Figure 1 shows the PRISMA flowchart of study selection. Given the limited literature base, a broad search strategy was adopted in an attempt to capture all possible studies (See eMethods for more details). A total of 15,990 records were identified from database searches. After the removal of duplicates, 11,355 records were screened, of which 508 documents were reviewed for eligibility by full text. A total of 20 studies were included, where 16 studies were included in the quantitative analysis [12, 13, 30–43] and an additional four studies were included in the qualitative synthesis due to insufficient data for quantitative synthesis [44–47]. Among the 16 studies included in the quantitative analysis, there was minimal missing outcome data, with only one timepoint of an outcome missing in a single study. Additionally, supplementary and/or raw data were received from eight of the 16 studies.

The characteristics and key findings of the 20 included studies are presented in Table 2 and eTable 3. Outcome measures and dependent variables for each study included in the quantitative synthesis are presented in eTables 4 and 5. Seventeen studies were double-blind, randomized, placebo-controlled cross-over designs and three were double-blind, randomized, placebo-controlled parallel-group trials. Of the eligible studies, 14 included healthy adult participants [12, 13, 30–36, 38, 39, 42, 46, 47]; one study included adults with social anxiety disorder [44]; one study was comprised of participants with psychosis [41]; one study was comprised of participants at high-risk of psychosis [45]; one study was participants with nicotine dependence [37]; one study included healthy adults with low and high Schizotypy Personality Questionnaire scores, but no clinically diagnosed schizophrenia or psychosis [40]; and one study included adults with chronic pain and fibromyalgia [43]. The majority of study populations were cannabis-naive or had few lifetime exposures. Only three studies included participants who had a recent history of occasional or frequent cannabis use [40, 42, 45]. Cannabinoids were primarily administered through an oral route (*N* = 14, 70%) or via vapourization (*N* = 5, 25%) alone, with one study administering both oral and vapourized cannabinoids (*N* = 1, 5%) [13]. Doses of oral CBD ranged from 15 mg to 4500 mg and from 12.5 to 400 mg for vapourized CBD. Doses of oral Δ9-THC ranged from 10 mg to 30 mg and vapourized doses ranged from 8 mg to 30 mg Δ9-THC. All included studies used a single-dose regimen. Qualitative findings are presented in the eResults.

**Table 2:** Study characteristics and key findings.

Study	Trial design	Country	Population	Age (years)	% M	N	Cannabis use behavior	Treatment(s) groups	Cannabinoid type	Route of administration	Outcome measure and assessment session post-treatment	Key findings
Arkell et al. [30]	Double-blind Randomized Placebo-controlled Cross-over	Netherlands	Healthy adults with occasional cannabis use	26.2 (2.6)	38	26	Use cannabis <2x/week in the past 12 months and >10 lifetime exposures	CBD, 13.75 mg THC, 13.75 mg THC, 13.75 mg + CBD, 13.75 PLA, 0 mg	Whole plant	Vapourization	Objective On-road driving (60 min duration) at 40–100, 240–300 min. DSST, DAT, PSAT at 5, 205 min. TOL at 5, 135 min Subjective VAS (sedation)at BL, 25, 120, 200, 240 min	No CBD-associated impaired performance reported
Arout et al. [31]	Double-blind Randomized Placebo-controlled Cross-over	United States of America	Healthy adults	32 (8)	47	17	Non-cannabis using participants Previous cannabis use history NR	CBD, 200 mg CBD, 400 mg CBD, 800 mg PLA, 0 mg	Isolate	Oral	Subjective VAS (alert), VAS (tired), VAS (stimulated) at BL, 30 min, and every 30 min until 360 min	No CBD-associated impaired performance reported
Bergamaschi et al., 2011	Double-blind Randomized Placebo-controlled Parallel-group	Brazil	adults with social anxiety disorder	CBD 24.6 (2.4) PLA 22.9 (2.4)	50	12	No cannabis use in the past year and ≤5 lifetime exposures	CBD, 600 mg PLA, 0 mg	Isolate	Oral	Subjective VAMS (sedation), VAMS (cognitive impairment) at BL, 80 min VAMS (sedation and cognitive impairment) were also assessed at 94 min, 107 min, 130 min, 150 min but these time points were not included in our analysis as they were taken post-stress test.	No CBD-associated impaired performance reported
Bhattacharyya et al. [32]	Double-blind Randomized Placebo-controlled Cross-over	England	Healthy adults	26.5	100	15	No cannabis use in the past month and <15 lifetime exposures	CBD, 600 mg THC, 10 mg PLA, 0 mg	Isolate	Oral	Objective VPAL at BL, 60, 120 min Subjective VAMS (sedation) at BL, 60, 120 min	No CBD-associated impaired performance reported
Bhattacharyya et al. [33]	Double-blind Randomized Placebo-controlled Cross-over	England	Healthy adults	26.5	100	15	No cannabis use in the past month and <15 lifetime exposures	CBD, 600 mg THC, 10 mg PLA, 0 mg	Isolate	Oral	Objective Visual oddball task between 60–120 min	Improved performance in outcome of reduced response latency for CBD compared to PLA
Bhattacharyya et al. [45]	Double-blind Randomized Placebo-controlled Parallel-group	England	Healthy adult controls (HC) + adults at clinical high risk (CHR) of psychosis	CHR-CBD 22.43 (4.95) CHR-PLA 25.35 (5.24)	CHR-CBD 63 CHR-PLA 41	15 (CBD) 16 (PLA)	No cannabis use within 96 hours of trial Some CHR participants reported chronic, occasional cannabis use (>1 X/week)	CBD, 600 mg PLA, 0 mg	Isolate	Oral	Objective VPAL (12 min duration) at BL, 180–240 min	No CBD-associated impaired performance reported
Bloomfield et al. [34]	Double-blind Randomized Placebo-controlled Cross-over	England	Healthy adults	24.1 (5)	40	15	No current or past use of CBD or cannabis	CBD, 600 mg PLA, 0 mg	Pure synthetic	Oral	Objective DST (forward, backward) at 320 min (*N* = 15) N-back (0-back, 1-back, 2-back) at 280 min (*N* = 13) Prose recall (immediate) at 275 min Prose recall (delayed) at 300 min (*N* = 15)	No CBD-associated impaired performance reported
Borgwardt et al. [35]	Double-blind Randomized Placebo-controlled Cross-over	England	Healthy adults	26.7 (5.7)	100	15	No cannabis use in the past month ≤15 lifetime exposures	CBD, 600 mg THC, 10 mg PLA, 0 mg	Isolate **NR - probably isolate but not clearly reported	Oral	Objective Go/No-Go task at 60 min	No CBD-associated impaired performance reported
Consroe et al. [36]	Double-blind Randomized Placebo-controlled Cross-over	Brazil	Healthy adults	21-33	60	10	No current cannabis use History of occasional recreational use (>1 X/week) several years prior to trial	CBD, 200 mg PLA, 0 mg	Isolate	Oral	Objective Cancellation test, differential aptitude test, time production task, and finger tap test at BL, 30, 60, 120, and 240 min Subjective Subjective drug reaction scale (perception of state, cognition, alertness and attention) BL, 30, 60, 120, and 240 min	No CBD-associated impaired performance reported
Hindocha et al. [37]	Double-blind Randomized Placebo-controlled Cross-over	England	Healthy adults with cigarette dependence	28.07 (8.66)	50	30	No cannabis use in the past month History of occasional recreational use (>1 X/month) ~100 days prior to trial	CBD, 800 mg PLA, 0 mg	Pure synthetic	Oral	Objective Go/No-Go task, Prose recall (immediate), N-back test, Prose recall (delayed) at 150 min	Go/No-go: Increased commission errors for CBD compared to placebo N-back and prose recall: No significant CBD-associated impaired performance reported
Hotz et al. [38]	Double-blind Randomized Placebo-controlled Cross-over	Switzerland	Healthy adults	22.26 (3.04)	50	34	No cannabis use in the past 7 days	CBD, 12.5 mg PLA, 0 mg	E-liquid	Vapourization	Objective Delayed free recall at 20 min N-back (0-back, 2-back) between 0–20 min Subjective VAS (fatigue) between 0–20 min	Delayed free recall: Enhanced episodic memory for CBD compared to placebo N-Back: No significant CBD-associated impaired performance in attention or working memory VAS: No worsening fatigue associated with CBD
McCartney et al. [39]	Double-blind Randomized Placebo-controlled Cross-over	Australia	Healthy adults	27.9 (7.0)	59	17	No cannabis use in the past 3 months	CBD, 15 mg CBD, 100 mg CBD, 1500 mg PLA, 0 mg	Pure Synthetic	Oral	Objective Simulated driving at 45–75 and 210–240 min DAT, DSST, PSAT at BL, 15–45, 180–210 min DRUID test at BL, 15–45, 180–210 min PVT at 75–90 and 240–260 min Subjective VAS (alert, sedated, sleepy) at BL, 15–45, 75–95, 140–150, 180–210, 240–260	Driving, DSST, PASAT, PVT, DRUID: No CBD-associated impaired performance. DAT: Higher 300 mg and 1500 mg dose of CBD improved tracking error compared to lower 15 mg CBD dose VAS alert and sedation: No difference between CBD and placebo, decreased alertness and increased sedation across sessions for both conditions* *Individual analysis indicated attributable to time not treatment effect
Morgan et al. [40]	Double-blind Randomized Placebo-controlled Cross-over	England	Healthy adults with Low or High Schizotypy (LS vs. HS) and Light or Heavy (L vs. H) Cannabis use	LS-L 21 (2.13) LS-H 21. 42 (1.62) HS-L 22.9 (2.02) HS-H 21.5 (1.38)	LS-L 75 LS-H 92 HS-L 59 HS-H 59	48 (47 for spatial 1-N back)	History of light (1–24 days per month) or heavy (25 + days per month) cannabis use No cannabis use in the past 24 hours	CBD, 16 mg THC, 8 mg THC, 8 mg + CBD, 16 mg PLA, 0 mg	Pure synthetic	Vapourization	Objective Prose recall (immediate) at BL and ~10 min Prose recall (delayed) at BL, 20 min N-back, fluency test, TMT-A, TMT-B at BL, ~10-20 min	Prose recall, N-back, and fluency: No CBD-associated impaired performance reported TMT: Significantly faster completion with CBD vs. placebo for TMT-A but not TMT-B
O’Neill et al. [41]	Double-blind Randomized Placebo-controlled Cross-over	England	Adults with psychosis (PSY)	PSY 27.73 (4.61)	PSY 66.7	13	≤10 lifetime cannabis exposures	PSY CBD, 16 mg PLA, 0 mg	Isolate	Oral	Objective VPAL (12 min duration) at 180 min	No CBD-associated impaired performance reported
Schoedel et al. [12]	Double-blind Randomized Placebo-controlled Cross-over	Canada	Healthy adults with polydrug use experience	37.7 (8.9)	72	43	≥10 non-therapeutic lifetime cannabis exposures ≥1 nontherapeutic cannabis exposures in the last 12 weeks prior to screening ≥1 non-therapeutic use of a CNS depressant in the last 12 weeks prior to screening	CBD, 750 mg CBD, 1500 mg CBD, 4500 mg THC, 10 mg THC, 30 mg Alprazolam 2 mg PLA, 0 mg	CBD Isolate (Epidiolex) THC Synthetic (Dronabinol)	Oral	Objective DAT, HVLT-R, DSST at BL, 60, 120, 180, 360, 480, 720, 1140 min Subjective VAS (alert vs. drowsy/sedative vs. stimulant) at BL, 30, 60, 90, 120, 150, 180, 240, 300, 360, 480, 600 min	DAT, HVLT, DSST: No CBD-associated impaired performance reported VAS sedation: Increased drowsiness with CBD compared to placebo but less than positive THC controls
Solowij et al. [42]	Double-blind Randomized Placebo-controlled Cross-over	Australia	Healthy adults with infrequent (infreq.) or frequent (freq.) cannabis use patterns	21 (18–51)	86.11	36	Infreq. 6-123 lifetime exposures Current cannabis use: ~0 days/month (range 0–5) for 0 years (range 0–4.5) Freq. 133-8000 lifetime exposures Current cannabis use: median of 10 days/month (range 2–28) and ≥1 use(s)/month for a median of 3 years (range 1.4-25.5)	CBD, 400 mg THC, 8 mg THC, 8 mg + CBD, 4 mg THC, 12 mg + CBD, 400 mg PLA, 0 mg	CBD Isolate (98% pure) THC Isolate (98% pure)	Vapourization	VAS (Drowsy) at 0, immediately after administration, and 55 min	CBD led to marginally greater drowsiness relative to PLA for infrequent users
Spindle et al. [13]	Double-blind Randomized Placebo-controlled Cross-over	United States	Healthy adults	31 (6)	50	18	1–3 lifetime CBD exposures but 69% had no previous CBD use No cannabis use ≥30 days prior to first experimental session Reported no cannabis use 148 (SD = 250) days prior to trial	CBD, 100 mg oral CBD, 100 mg vape CBD, 100 mg + THC, 3.7 mg vape PLA, 0 mg oral PLA, 0 mg vape	CBD Synthetic isolate (vape) Pure isolate (oral) CBD + THC Whole plant	Vapourization + oral	Objective DSST, PSAT, DAT at BL, 0, 30, 60*, 90, 120, 180, 240, 300, 420 min Subjective VAS (sleepy), VAS (alert), VAS (trouble with memory) at 0, 30, 60*, 90, 120, 180, 240, 300, 420 min *The 60 min time point occurred immediately after the vapor dose administration period was completed. At BL participants received oral CBD or PLA.	DSST, PSAT, DAT and VAS: No significant CBD-associated impaired performance. VAS: CBD-dominant cannabis had greater rating of sedation than vapourized pure, but not significantly different from placebo
van de Donk et al. [43]	Double-blind Randomized Placebo-controlled Cross-over	Netherlands	Adults with chronic pain + fibromyalgia	39 (13)	0	20	No recent cannabis use	CBD, 18.4 mg + THC, <1 mg CBD, 12.8 mg + THC 13.4 mg CBD, <3 mg +THC, 22.4 mg PLA	Whole plant	Vapourization	Subjective Bond and Lader VAS (alert-drowsy) at BL, 30, 60, 90, 120, 150, 180 min	VAS: No CBD-associated impaired performance reported
Winton-Brown et al. [46]	Double-blind Randomized Placebo-controlled Cross-over	England	Healthy adults	26.7 (5.7)	100	14	No cannabis use in the past month >1 but <15 lifetime exposures	CBD, 600 mg THC, 10 mg PLA, 0 mg	NR	Oral	Subjective VAMS (mental sedation)	No CBD-associated impaired performance reported
Woelfl et al. [47]	Double-blind Randomized Placebo-controlled Parallel-group	Germany	Healthy adults	Median: ~25.5 Range: (19–36)	100	15	No cannabis use in the 6 months >1 but <10 lifetime exposures	CBD, 800 mg + PLA, 0 mg CBD, 800 mg + THC, 20 mg PLA, 0 mg + THC, 20 mg PLA, 0 mg + PLA, 0 mg	Isolate	Oral	Objective DSST, Letter-number sequencing task, d2 Test of Attention at BL and ~205 min	No CBD-associated impaired performance reported

*BL* baseline, *CBD* cannabidiol, *DAT* divided attention task, *DST* digit span task, *DSST* digit symbol substitution task, *HVLT-R* hopkins verbal learning test—revised, *PLA* placebo, *PSAT* Paced serial addition task, *PVT* psychomotor vigilance task, *THC* tetrahydrocannabinol, *TMT-A* trail making test part a, *TMT-B* trail making test part b, *TOL* tower of London, *VAS* visual analog scale, *VAMS* visual analog mood scale, *VPAL* verbal paired associate learning.

### Quantitative findings

#### Omnibus meta-analysis of peak performance effects of acute CBD exposure compared to placebo

The omnibus RVE meta-analysis indicated a significant, but small effect size for impaired performance following acute CBD consumption compared to placebo (Hedges’ *g* = 0.122, 95% CI: 0.023–0.221, *p* = 0.019). Moderate heterogeneity was observed among studies (*I*^2^ = 38.24%). A consistent omnibus estimate was observed when collapsing effect sizes into a single average estimate for each study, Hedges’ *g* = 0.113, 95% CI: 0.014–0.212, *p* = 0.026. Model results are presented in Table 3.

**Table 3:** Summary of primary meta-analytic findings.

Measure	Effect estimates (*n*)	Coefficient Estimate of Robust Variance Estimation (RVE) Meta-Analysis	Hedges’ *g*
*n* = 354			
Studies = 16			
Effect Size			
Omnibus SMD (95% CI, *I*^*2*^)	154	0.122 (0.023–0.221, *I*^*2*^ = 38.24%)*	0.122
Moderators (95% CI)			
Measure type			
Objective^a^	122	0.048 (−0.028–0.124)	0.048
Subjective	33	0.24* (0.028–0.455)	0.288
Cognitive functions			
Subjective^a^ sedation/tired	22	0.329 (0.089–0.570)	0.329
Episodic memory	15	−0.263* (−0.499 to −0.027)	0.066
Working memory	18	−0.303* (−0.526 to −0.08)	0.026
Divided attention	38	NA	
Driving	8	NA	
Executive function	3	NA	
Information processing	25	−0.260 (−0.655 to 0.1347)	n.s.
Subjective alertness	9	NA	
Dose			
Intercept	144	0.116 (0.018–0.215)	n.s.
Dose (Continuous)	144	0 (0–0)	n.s.
Route of administration			
Oral^a^	112	0.155 (−0.106 to 0.417)	n.s.
Inhaled	43	−0.056 (−0.291 to 0.179)	n.s.

Moderator values represent the meta-regression results and are described by the coefficient estimates. Effect estimates (Hedges’ *g*) are displayed for significant covariates. The highest dose of 4500 mg CBD administered in one study was excluded from this analysis in order to reduce data skewness and allow for model interpretability.

*NA* insufficient degrees of freedom to interpret results, *n.s.* not significant, *SMD* standardized mean difference.

*p < 0.05.

^a^Reference group (intercept).

#### Moderator analyses

Results of moderator analyses are presented in Table 3. A significant moderator effect was observed for measure type. This effect reflected larger mean differences between CBD and placebo when subjective measures were used (Hedges’ *g*_Subjective_ = 0.288 versus Hedges’ *g*_Objective_ = 0.048). Objective measures were not found to be significantly different than 0 when changed to the reference group to test the significance of the intercept.

A significant moderation effect was observed for cognitive function. This reflected a significantly larger mean difference for measures of subjective sedation/tiredness (Hedges’ *g* = 0.329) compared to episodic memory (Hedges’ *g* = 0.066) and working memory (Hedges’ *g* = 0.026). Information processing measures were not observed to have a significantly different mean difference compared to subjective sedation/tiredness measures (*p* = 0.157). However, only subjective sedation/tiredness was observed to be significantly different from 0. Comparisons to measures of divided attention, driving, executive function and subjective alertness could not be made due to insufficient degrees of freedom.

CBD dose and route of administration were not significant moderators (ps > 0.5).

#### Comparisons to Δ9-THC

Two secondary analyses were conducted to assess the difference in peak performance effect between CBD and Δ9-THC (Table 4). The first analysis compared peak mean difference in performance measures between cannabis and placebo, with cannabinoid (CBD or Δ9-THC) as a moderator. Eight studies that had a CBD and a Δ9-THC arm were included. Cannabinoid type was a significant moderator of effect sizes. This effect reflected larger mean differences between Δ9-THC and placebo (Hedges’ *g* = 0.356, 95% CI: 0.059–0.398, *p* = 0.016) compared to CBD and placebo (Hedges’ *g* = 0.128).

**Table 4:** Summary of secondary meta-analyses.

Measure	Effect estimates (*n*)	Coefficient Estimate of Robust Variance Estimation (RVE) Meta-Analysis	Hedge’s *g*
*n* = 200			
Studies = 8			
Effect Size comparison between CBD-PLA vs Δ9-THC-PLA			
Omnibus SMD (95% CI, *I*^*2*^)	102	0.237 (0.019–0.456, *I*^*2*^ = 69%)**	0.237
Cannabinoid type moderation (95% CI)			
CBD^a^	62	0.128 (−0.059–0.316)	0.128
Δ9-THC	40	0.228 (0.059–0.398)**	0.356
Effect Size comparison between Δ9-THC vs CBD			
Omnibus SMD (95% CI, *I*^*2*^)	66	0.416 (95% CI 0.017–0.816, *I*^*2*^ = 83.77%)**	0.416

Moderator values represent the meta-regression results and are described by the coefficient estimates. Effect estimates are displayed as Hedges’ *g*. Larger positive effect estimates indicate a greater impairing effect.

*PLA* placebo, *SMD* standardized mean difference.

**p < 0.01.

^a^Reference group (intercept).

The second analysis provided a direct comparison of the peak mean difference in performance measures between Δ9-THC consumption compared to CBD consumption. Eight studies that had a CBD and Δ9-THC arm were included. The omnibus RVE meta-analysis indicated a significantly greater effect on performance for Δ9-THC compared to CBD. This effect reflected a moderate effect size for impaired performance following Δ9-THC consumption compared to CBD (Hedges’ *g* = 0.416, 95% CI: 0.017–0.816, *p* = 0.043).

### Quality of evidence

The quality of available evidence was deemed moderate-to-high (See eFig. 1 and eResults). Of the 20 clinical trials analyzed, three (15%) were deemed to have an overall ‘low risk’ of bias, 16 (76%) were assessed as having ‘some concerns’, and one (5%) was identified as ‘high risk’ of bias.

Egger’s test for funnel plot asymmetry was not statistically significant, consistent with the funnel plot visual (See eFig. 2 and eResults).

## Discussion

The results of this meta-analysis indicate that acute CBD consumption had a small but statistically significant effect on performance as assessed by all outcomes in aggregate, compared to placebo. Moderator analyses revealed this effect was significant only for subjective ratings of sedation/drowsiness, and no significant effects were observed for objective task performance on domains including memory, psychomotor ability, driving performance, information processing, attention, or higher order cognitive functioning. Dose and route of administration were not significant moderators in this analysis. As expected, acute doses of Δ9-THC produced significantly greater impaired performance than CBD relative to placebo and in direct comparison to CBD under the same experimental conditions. It is important to note that this sample was composed of primarily naive or infrequent cannabis users. It is unknown if these findings would translate to individuals with consistent cannabis product use, generally, or CBD use, specifically (e.g., medical cannabis patients). Additionally, the small, statistically significant effect size for the primary comparison of performance on cognitive and psychomotor measures between CBD to placebo may not translate to functional impairment, particularly given that these differences were limited to subjective feelings of sedation or tiredness.

This evidence synthesis supports that acute CBD consumption does not negatively impact neurocognitive function, as assessed by objective neurocognitive measures, consistent with findings from earlier trials and reviews [11, 48, 49]. It is important to note that these findings are from a sample of primarily infrequent cannabis consumers and may not represent the actual population of individuals who use CBD chronically. Infrequent cannabis consumers would most likely have the highest risk of impairment compared to individuals who use CBD chronically. Additionally, this sample was primarily in healthy adults. The effect of CBD may be different in different clinical populations. The small effect of subjective sedation noted in the current study has been reported inconsistently within previous literature. Somnolence and sedation are noted as potential side effects in Epidiolex prescribing information [50]. However, it has been proposed that CBD-related sedation in the context of the treatment of epilepsy may be due to drug interactions rather than CBD itself [51, 52].

Discrepancies between subjective and objective indicators of impairment have been noted previously. Some evidence suggests that people who use cannabis may overestimate their level of sedation and other indicators of impairment [53, 54], while others may compensate for expected impairment-related effects [55]. Drug expectancy may also contribute to this phenomenon. The expectation of receiving a certain drug can produce subjective and behavioral effects similar or opposite to those related to the drug, even in the absence of the drug itself. Such expectations can be formed by verbal information about the content and supposed effects of the drug, prior experience, and observational learning [56]. Metrik et al. [55, 57] have shown that the expectancy of receiving Δ9-THC produces greater subjective effects, including euphoria and sedation. CBD expectancy may also impact subjective and drug effect ratings [58]. Given that cannabis expectancy seems to affect self-reported reactions and drug responses, this calls into question the level of functional impairment associated with the small effect size obtained from this synthesis.

As expected, Δ9-THC produced significantly higher magnitudes of impaired performance compared to CBD. This adds validation for detecting and examining impaired cognitive and psychomotor performance for CBD and THC using the same experiments and designs. However, the question of whether concurrent CBD and Δ9-THC consumption increases or decreases the magnitude of impairment remains. Many cannabis products contain both CBD and Δ9-THC, including whole-plant CBD-dominant products. Additionally, CBD may be co-administered with Δ9-THC preparations with the expectation that CBD can ameliorate Δ9-THC-related cognitive impairment, anxiety, and sedation while also offering a range of therapeutic benefits [59–61]. Evidence from both experimental and naturalistic studies suggest that the addition of CBD to Δ9-THC produces differential dose-dependent effects, which may depend on the ratio of CBD:Δ9-THC and route of administration [30, 42, 43, 62, 63]. One study found that low-dose vapourized CBD (4 mg) enhanced impairment relative to Δ9-THC (8 mg) alone, whereas high-dose CBD (400 mg) reduced impairment across objective and subjective measures [42]. Other studies have reported that vapourized Δ9-THC/CBD-equivalent cannabis (13.75 mg Δ9-THC + 13.75 mg CBD) is no less impairing than Δ9-THC-dominant cannabis (13.75 mg Δ9-THC), and in some cases CBD may actually exacerbate Δ9-THC-induced acute impairment, as measured by psychomotor assessments and simulated driving performance [47, 62]. Pharmacokinetic data from the available research has also shown that peak plasma concentrations of Δ9-THC appear to be higher when CBD is co-administered [30, 43, 62], although several studies have also found no evidence of changes [64–66]. CBD can inhibit the metabolism of Δ9-THC and other drugs, and these interactions are more likely to occur after oral ingestion of CBD than with inhalation [67–70]. Thus, it is imperative to consider the potential for CBD to increase impairment when combined with other drugs, even if acute doses of CBD alone are not associated with functional impairment in controlled research studies.

The majority of participants in the current investigation were naive to cannabis or had few lifetime exposures. It has previously been observed that people who regularly use cannabis experience less cannabis-associated impairment compared to those with occasional use [9, 71]. As such, it is unknown how these findings would translate to populations with more frequent cannabis use (e.g., medical cannabis patients). However, it could be predicted that the small effect on subjective sedation observed in the current study may be diminished with frequent CBD use, in line with what has been observed in studies assessing Δ9-THC-associated impairment in frequent cannabis users [9, 72]. Further, some evidence suggests that CBD may improve cognitive function with prolonged use [73].

### Future directions

The available literature on the acute performance effects of CBD consumption only allowed for assessment of performance in certain domains of cognitive function and in certain contexts of use (e.g., naive to cannabis consumption). Of key importance, there is a need to examine the impact of frequent, long-term CBD use on neurocognitive function to examine if tolerance diminishes the observed effect. Particularly for common safety sensitive tasks completed by the general population, such as driving, to gain a more robust picture of real-world risk. Finally, the majority of the trials in this study used CBD isolate products. In the real-world, full spectrum CBD-dominant products (which include other major and minor cannabinoids [including low levels of Δ9-THC] and terpenes), balanced CBD:Δ9-THC products, and lower CBD to Δ9-THC ratio products are commonly used. Effects on neurocognitive performance associated with these products should be further investigated as other cannabinoids and terpenes may contribute to impairing effects.

### Limitations

This meta-analysis had several limitations. There was insufficient data, due to the sparse number of studies that included frequent cannabis users, to examine the potential difference between infrequent and frequent cannabis users. As such, these findings may not translate to populations who consistently use CBD. Additionally, although moderation analyses were conducted to assess variability, there are undoubtedly other variables that may impact an individual’s magnitude or risk of impaired neurocognitive performance (e.g., comorbidities, concomitant medications) that were not addressed in the included studies.

## Conclusion

This meta-analysis suggests acute CBD consumption may be associated with a small increase in subjective sedation compared to placebo in infrequent cannabis users, but does not significantly impact performance across a range of cognitive domains. These results are consistent with previous evidence supporting that CBD consumption does not impact neurocognitive function. As such, acute use of CBD in the absence of THC or other drugs is unlikely to lead to functional impairment. Further research is warranted to investigate the risk of impaired neurocognitive function with daily CBD consumption, in addition to assessing performance in alternative domains.

### Supplementary information

Supplementary Material

eTable 5. Dependent variables by study

## Data Availability

Data related to this manuscript will be made available upon request.
