# Supplementary material for: Does acute cannabidiol (CBD) use impair performance? A meta-analysis and comparison with placebo and delta-9-tetrahydrocannabinol (THC)
Source: Neuropsychopharmacology. 2024 Mar 25;49(9):1425–36. doi: 10.1038/s41386-024-01847-w (PMC11251190; doi:10.1038/s41386-024-01847-w)
Supplement: Supplementary file 1 — Supplementary Material [file 41386_2024_1847_MOESM1_ESM.docx]

**Online-only Supplemental Materials**

**Table of Contents**

1. **eMethods**
2. **eTable 1. Search Strategies**
3. **eTable 2. PICOS statement**
4. **eResults**
5. **eTable 3. Key characteristics of all included trials**
6. **eTable 4. Studies by outcome measure included in the quantitative synthesis**
7. **eFigure 1. Risk of Bias assessment**
8. **eFigure 2. Funnel plot**
9. **eMethods**

**Search strategy**

Given the limited literature on acute CBD impairment, a very broad strategy was adopted to try and capture all possible studies. Minimal restrictions were used as to not miss studies that were not indexed in databases by certain MeSH terms/criteria. For example, the search was not constrained to “humans only” as not all human trials are indexed as human, lending to a very large number of animal studies that were excluded early on. These strategies were adopted in collaboration with a medical librarian to make sure we captured all possible trials within our search. As a result, this inclusive strategy resulted in many records that were not eligible, but we believe this was a preferred approach to ensure a more thorough search of a limited literature base.

Further, the original search included Δ9-THC impairment studies without a CBD arm for the secondary positive control analysis. However, this was then limited to only include studies with both a CBD and Δ9-THC arm to reduce heterogeneity in comparing CBD and Δ9-THC-related impairment. This decision was made after full text screening during preliminary data extraction, prior to any results being extracted or analyses being run.

**Data extraction**

Eligible effect estimates for the peak mean difference in studies with multiple time points were constrained to 0-120 minutes post-inhaled cannabis and 30-240 minutes post-oral cannabis consumption given the pharmacokinetics of each route of administration [1–3].

**Moderator variables**

*Measure type*. Measure type was coded as a dichotomous variable representing subjective (k = 33) versus objective (k = 122) measures of impairment.

*Cognitive function*. Cognitive function was coded as a categorical variable based on the cognitive function/domain being assessed by the study measure (Table 1). Categorizations of measures by cognitive function/domain were based on previous meta-analyses [4,5]. Variable levels were limited to function domains that were assessed by > 2 studies: subjective sedation/tiredness (k = 22), divided attention (k = 38), driving (k = 8), episodic memory (k = 15), executive function (k = 3), information processing (k = 25), subjective alertness (k = 9), and working memory (k = 18).

*CBD dose*. CBD dose was included as a continuous variable. The highest dose of 4500 mg CBD administered in one study was excluded from this analysis in order to reduce data skewness and allow for model interpretability.

*Route of administration*. Route of administration was coded as a dichotomous variable representing oral

(k = 122) versus inhaled (k = 43) routes of administration. The inhalation method used in included trials was

vapourization.

1. **eTable 1. Search Strategies**

**eTable 1. Search Strategies**

| **Line** | **Search term** |
| --- | --- |
| **EMBASE** | |
| 1 | cannabidiol/ |
| 2 | CBD.mp. [mp=title, abstract, heading word, drug trade name, original title, device manufacturer, drug manufacturer, device trade name, keyword heading word, floating subheading word, candidate term word] |
| 3 | Cannabidiol.mp. [mp=title, abstract, heading word, drug trade name, original title, device manufacturer, drug manufacturer, device trade name, keyword heading word, floating subheading word, candidate term word] |
| 4 | Epidiolex.mp. [mp=title, abstract, heading word, drug trade name, original title, device manufacturer, drug manufacturer, device trade name, keyword heading word, floating subheading word, candidate term word] |
| 5 | Nabidiokex.mp. [mp=title, abstract, heading word, drug trade name, original title, device manufacturer, drug manufacturer, device trade name, keyword heading word, floating subheading word, candidate term word] |
| 6 | Epidyolex.mp. [mp=title, abstract, heading word, drug trade name, original title, device manufacturer, drug manufacturer, device trade name, keyword heading word, floating subheading word, candidate term word] |
| 7 | hemp.mp. |
| 8 | 1 or 2 or 3 or 4 or 5 or 6 or 7 |
| 9 | cannabinoid/ |
| 10 | exp cannabis/ |
| 11 | dronabinol/ |
| 12 | tetrahydrocannabinol/ |
| 13 | nabiximols/ |
| 14 | cannabi*.mp. [mp=title, abstract, heading word, drug trade name, original title, device manufacturer, drug manufacturer, device trade name, keyword heading word, floating subheading word, candidate term word] |
| 15 | marijuana.mp. [mp=title, abstract, heading word, drug trade name, original title, device manufacturer, drug manufacturer, device trade name, keyword heading word, floating subheading word, candidate term word] |
| 16 | marihuana.mp.[mp=title, abstract, heading word, drug trade name, original title, device manufacturer, drug manufacturer, device trade name, keyword heading word, floating subheading word, candidate term word] |
| 17 | tetrahydrocannabinol.mp, [mp=title, abstract, heading word, drug trade name, original title, device manufacturer, drug manufacturer, device trade name, keyword heading word, floating subheading word, candidate term word] |
| 18 | THC.mp. [mp=title, abstract, heading word, drug trade name, original title, device manufacturer, drug manufacturer, device trade name, keyword heading word, floating subheading word, candidate term word] |
| 19 | Sativex.mp. [mp=title, abstract, heading word, drug trade name, original title, device manufacturer, drug manufacturer, device trade name, keyword heading word, floating subheading word, candidate term word] |
| 20 | dronabinol.mp. [mp=title, abstract, heading word, drug trade name, original title, device manufacturer, drug manufacturer, device trade name, keyword heading word, floating subheading word, candidate term word] |
| 21 | 9 or 10 or 11 or 12 or 13 or 14 or 15 or 16 or 17 or 18 or 19 or 20 |
| 22 | intoxication/ or drug intoxication/ |
| 23 | balance impairment/ |
| 24 | exp memory/ |
| 25 | exp attention/ |
| 26 | psychomotor performance/ or psychomotor activity/ or task performance/ |
| 27 | motor performance |
| 28 | cognitive/ or executive function/ |
| 29 | verbal learning/ or paired associate learning/ |
| 30 | discrimination learning/ |
| 31 | associative learning/ or paired associate learning/ |
| 32 | mental performance/ or mental load/ or mental task/ |
| 33 | psychomotor activity/ or driving ability/ |
| 34 | exp reaction time/ or auditory reaction time/ or brake reaction time/ or motor reaction time/ or stop signal reaction time/ or visual reaction time/ |
| 35 | cogniti*.mp. [mp=title, abstract, heading word, drug trade name, original title, device manufacturer, drug manufacturer, device trade name, keyword heading word, floating subheading word, candidate term word] |
| 36 | impairm*.mp. [mp=title, abstract, heading word, drug trade name, original title, device manufacturer, drug manufacturer, device trade name, keyword heading word, floating subheading word, candidate term word] |
| 37 | psychomotor.mp. [mp=title, abstract, heading word, drug trade name, original title, device manufacturer, drug manufacturer, device trade name, keyword heading word, floating subheading word, candidate term word] |
| 38 | attention.mp. [mp=title, abstract, heading word, drug trade name, original title, device manufacturer, drug manufacturer, device trade name, keyword heading word, floating subheading word, candidate term word] |
| 39 | memory.mp. [mp=title, abstract, heading word, drug trade name, original title, device manufacturer, drug manufacturer, device trade name, keyword heading word, floating subheading word, candidate term word] |
| 40 | reaction time.mp, [mp=title, abstract, heading word, drug trade name, original title, device manufacturer, drug manufacturer, device trade name, keyword heading word, floating subheading word, candidate term word] |
| 41 | coordinat*.mp. [mp=title, abstract, heading word, drug trade name, original title, device manufacturer, drug manufacturer, device trade name, keyword heading word, floating subheading word, candidate term word] |
| 42 | intoxic*.mp. [mp=title, abstract, heading word, drug trade name, original title, device manufacturer, drug manufacturer, device trade name, keyword heading word, floating subheading word, candidate term word] |
| 43 | driving.mp. [mp=title, abstract, heading word, drug trade name, original title, device manufacturer, drug manufacturer, device trade name, keyword heading word, floating subheading word, candidate term word] |
| 44 | task switching.mp. [mp=title, abstract, heading word, drug trade name, original title, device manufacturer, drug manufacturer, device trade name, keyword heading word, floating subheading word, candidate term word] |
| 45 | processing speed.mp. [mp=title, abstract, heading word, drug trade name, original title, device manufacturer, drug manufacturer, device trade name, keyword heading word, floating subheading word, candidate term word] |
| 46 | or/22-45 |
| 47 | 8 or 21 |
| 48 | 46 and 47 |
| 49 | (Randomized controlled trial/ or Controlled clinical study/ or random$.ti,ab. or randomization/ or intermethod comparison/ or placebo.ti,ab. or (compare or compared or comparison).ti. or ((evaluated or evaluate or evaluating or assessed or assess) and (compare or compared or comparing or comparison)).ab. or (open adj label).ti,ab. or ((double or single or doubly or singly) adj (blind or blinded or blindly)).ti,ab. or double blind procedure/ or parallel group$1.ti,ab. or (crossover or cross over).ti,ab. or ((assign$ or match or matched or allocation) adj5 (alternate or group$1 or intervention$1 or patient$1 or subject$1 or participant$1)).ti,ab. or (assigned or allocated).ti,ab. or (controlled adj7 (study or design or trial)).ti,ab. or (volunteer or volunteers).ti,ab. or human experiment/ or trial.ti.) not (((random$ adj sampl$ adj7 ("cross section$" or questionnaire$1 or survey$ or database$1)).ti,ab. not (comparative study/ or controlled study/ or randomi?ed controlled.ti,ab. or randomly assigned.ti,ab.)) or (Cross-sectional study/ not (randomized controlled trial/ or controlled clinical study/ or controlled study/ or randomi?ed controlled.ti,ab. or control group$1.ti,ab.)) or (((case adj control$) and random$) not  randomi?ed controlled).ti,ab. or (Systematic review not (trial or study)).ti. or (nonrandom$ not random$).ti,ab. or "Random field$".ti,ab. or (random cluster adj3 sampl$).ti,ab. or ((review.ab. and review.pt.) not trial.ti.) or ("we searched".ab. and (review.ti. or review.pt.)) or "update review".ab. or (databases adj4 searched).ab. or ((rat or rats or mouse or mice or swine or porcine or murine or sheep or lambs or pigs or piglets or rabbit or rabbits or cat or cats or dog or dogs or cattle or bovine or monkey or monkeys or trout or marmoset$1).ti. and animal experiment/) or (Animal experiment/ not (human experiment/ or human/))) |
| 50 | 48 and 49 |
| **Medline** | |
| 1 | Cannabidiol/ |
| 2 | CBD.mp. [mp=title, book title, abstract, original title, name of substance word, subject heading word, floating sub-heading word, keyword heading word, organism supplementary concept word, protocol supplementary concept word, rare disease supplementary concept word, unique identifier, synonyms] |
| 3 | Cannabidiol.mp. [mp=title, book title, abstract, original title, name of substance word, subject heading word, floating sub-heading word, keyword heading word, organism supplementary concept word, protocol supplementary concept word, rare disease supplementary concept word, unique identifier, synonyms] |
| 4 | Epidiolex.mp. [mp=title, book title, abstract, original title, name of substance word, subject heading word, floating sub-heading word, keyword heading word, organism supplementary concept word, protocol supplementary concept word, rare disease supplementary concept word, unique identifier, synonyms] |
| 5 | Epidyolex.mp. [mp=title, book title, abstract, original title, name of substance word, subject heading word, floating sub-heading word, keyword heading word, organism supplementary concept word, protocol supplementary concept word, rare disease supplementary concept word, unique identifier, synonyms] |
| 6 | hemp.mp. [mp=title, book title, abstract, original title, name of substance word, subject heading word, floating sub-heading word, keyword heading word, organism supplementary concept word, protocol supplementary concept word, rare disease supplementary concept word, unique identifier, synonyms] |
| 7 | 1 or 2 or 3 or 4 or 5 or 6 |
| 8 | exp Cannabinoids/ |
| 9 | Cannabis/ |
| 10 | cannabi*.mp. [mp=title, book title, abstract, original title, name of substance word, subject heading word, floating sub-heading word, keyword heading word, organism supplementary concept word, protocol supplementary concept word, rare disease supplementary concept word, unique identifier, synonyms] |
| 11 | dronabinol.mp. [mp=title, book title, abstract, original title, name of substance word, subject heading word, floating sub-heading word, keyword heading word, organism supplementary concept word, protocol supplementary concept word, rare disease supplementary concept word, unique identifier, synonyms] |
| 12 | marijuana.mp. [mp=title, book title, abstract, original title, name of substance word, subject heading word, floating sub-heading word, keyword heading word, organism supplementary concept word, protocol supplementary concept word, rare disease supplementary concept word, unique identifier, synonyms] |
| 13 | marihuana.mp. [mp=title, book title, abstract, original title, name of substance word, subject heading word, floating sub-heading word, keyword heading word, organism supplementary concept word, protocol supplementary concept word, rare disease supplementary concept word, unique identifier, synonyms] |
| 14 | tetrahydrocannabinol.mp. [mp=title, book title, abstract, original title, name of substance word, subject heading word, floating sub-heading word, keyword heading word, organism supplementary concept word, protocol supplementary concept word, rare disease supplementary concept word, unique identifier, synonyms] |
| 15 | THC.mp. [mp=title, book title, abstract, original title, name of substance word, subject heading word, floating sub-heading word, keyword heading word, organism supplementary concept word, protocol supplementary concept word, rare disease supplementary concept word, unique identifier, synonyms] |
| 16 | Sativex.mp. [mp=title, book title, abstract, original title, name of substance word, subject heading word, floating sub-heading word, keyword heading word, organism supplementary concept word, protocol supplementary concept word, rare disease supplementary concept word, identifier, synonyms] |
| 17 | 8 or 9 or 10 or 11 or 12 or 13 or 14 or 15 or 16 |
| 18 | exp Neuropsychological Tests/ |
| 19 | Cognitive Dysfunction/ |
| 20 | “Drug-Related Side Effects and Adverse Reactions”/ |
| 21 | cognition/ or Attention/ or comprehension/ or executive function/ or learning/ or association/ or exp memory/ or paired-associatelearning/ or perception/ or auditory perception/ or visual perception/ or decision making/ or exp psychomotor performance/ or Neuropsychological Tests/ or reaction time/ or reflex/ or postural balance/ |
| 22 | exp Attention/ |
| 23 | motor activity/ or multitasking behaviour/ |
| 24 | “Task Performance and Analysis”/ |
| 25 | automobile driving/ or driving under the influence/ |
| 26 | cogniti*.mp. [mp=title, book title, abstract, original title, name of substance word, subject heading word, floating sub-heading word, keyword heading word, organism supplementary concept word, protocol supplementary concept word, rare disease supplementary concept word, unique identifier, synonyms] |
| 27 | impair*.mp. [mp=title, book title, abstract, original title, name of substance word, subject heading word, floating sub-heading word, keyword heading word, organism supplementary concept word, protocol supplementary concept word, rare disease supplementary concept word, unique identifier, synonyms] |
| 28 | perform*.mp. [mp=title, book title, abstract, original title, name of substance word, subject heading word, floating sub-heading word, keyword heading word, organism supplementary concept word, protocol supplementary concept word, rare disease supplementary concept word, unique identifier, synonyms] |
| 29 | psychomotor.mp. [mp=title, book title, abstract, original title, name of substance word, subject  heading word, floating sub-heading word, keyword heading word, organism supplementary  concept word, protocol supplementary concept word, rare disease supplementary concept word,  unique identifier, synonyms] |
| 30 | attention.mp. [mp=title, book title, abstract, original title, name of substance word, subject  heading word, floating sub-heading word, keyword heading word, organism supplementary  concept word, protocol supplementary concept word, rare disease supplementary concept word,  unique identifier, synonyms] |
| 31 | motor.mp [mp=title, book title, abstract, original title, name of substance word, subject  heading word, floating sub-heading word, keyword heading word, organism supplementary  concept word, protocol supplementary concept word, rare disease supplementary concept word,  unique identifier, synonyms] |
| 32 | memory.mp. [mp=title, book title, abstract, original title, name of substance word, subject  heading word, floating sub-heading word, keyword heading word, organism supplementary  concept word, protocol supplementary concept word, rare disease supplementary concept word,  unique identifier, synonyms] |
| 33 | reaction time.mp. [mp=title, book title, abstract, original title, name of substance word, subject  heading word, floating sub-heading word, keyword heading word, organism supplementary  concept word, protocol supplementary concept word, rare disease supplementary concept word,  unique identifier, synonyms] |
| 34 | coordinat*.mp. [mp=title, book title, abstract, original title, name of substance word, subject  heading word, floating sub-heading word, keyword heading word, organism supplementary  concept word, protocol supplementary concept word, rare disease supplementary concept word,  unique identifier, synonyms] |
| 35 | intoxic*.mp. [mp=title, book title, abstract, original title, name of substance word, subject heading word, floating sub-heading word, keyword heading word, organism supplementary concept word, protocol supplementary concept word, rare disease supplementary concept word, unique identifier, synonyms] |
| 36 | driving.mp. [mp=title, book title, abstract, original title, name of substance word, subject heading word, floating sub-heading word, keyword heading word, organism supplementary concept word, protocol supplementary concept word, rare disease supplementary concept word, unique identifier, synonyms] |
| 37 | task switching.mp [mp=title, book title, abstract, original title, name of substance word, subject heading word, floating sub-heading word, keyword heading word, organism supplementary concept word, protocol supplementary concept word, rare disease supplementary concept word, unique identifier, synonyms] |
| 38 | processing speed.mp. [mp=title, book title, abstract, original title, name of substance word, subject heading word, floating sub-heading word, keyword heading word, organism supplementary concept word, protocol supplementary concept word, rare disease supplementary concept word, unique identifier, synonyms] |
| 39 | learn*.mp. [mp=title, book title, abstract, original title, name of substance word, subject heading word, floating sub-heading word, keyword heading word, organism supplementary concept word, protocol supplementary concept word, rare disease supplementary concept word, unique identifier, synonyms] |
| 40 | 7 or 17 |
| 41 | 18 or 19 or 20 or 21 or 22 or 23 or 24 or 25 or 26 or 27 or 28 or 29 or 30 or 31 or 32 or 33 or 34 oe 35 or 36 or 37 or 38 or 39 |
| 42 | 40 and 41 |
| 43 | ((randomized controlled trial or controlled clinical trial).pt. or randomized.ab. or randomised.ab. or placebo.ab. or drug therapy.fs. or randomly.ab. or trial.ab. or groups.ab.) not (exp animals/ not humans.sh.) |
| 44 | 42 and 43 |
| 45 | limit 44 to yr=”2022 -Current” |
| **AMED (Allied and Complementary Medicine)** | |
| 1 | CBD.mp. [mp=abstract, heading words, title] |
| 2 | Cannabidiol.mp. [mp=abstract, heading words, title] |
| 3 | Epidiolex.mp. [mp=abstract, heading words, title] |
| 4 | Nabidiolex.mp. [mp=abstract, heading words, title] |
| 5 | Epidyolex.mp. [mp=abstract, heading words, title] |
| 6 | 1 or 2 or 3 or 4 or 5 |
| 7 | cognit*.mp. [mp=abstract, heading words, title] |
| 8 | impair*.mp. [mp=abstract, heading words, title] |
| 9 | perform*.mp. [mp=abstract, heading words, title] |
| 10 | psychomotor*.mp. [mp=abstract, heading words, title] |
| 11 | attention*.mp. [mp=abstract, heading words, title] |
| 12 | motor.mp. [mp=abstract, heading words, title] |
| 13 | memory.mp. [mp=abstract, heading wHords, title] |
| 14 | reaction time.mp. [mp=abstract, heading words, title] |
| 15 | coordinat*.mp. [mp=abstract, heading words, title] |
| 16 | concentrat*.mp. [mp=abstract, heading words, title] |
| 17 | intoxic*.mp. [mp=abstract, heading words, title] |
| 18 | driving.mp. [mp=abstract, heading words, title] |
| 19 | task switching.mp. [mp=abstract, heading words, title] |
| 20 | processing speed.mp. [mp=abstract, heading words, title] |
| 21 | 7 or 8 or 9 or 10 or 11 or 12 or 13 or 14 or 15 or 16 or 17 or 18 or 19 or 20 |
| 22 | 6 and 21 |
| 23 | cannabi*.mp. [mp=abstract, heading words, title] |
| 24 | marijuana.mp. [mp=abstract, heading words, title] |
| 25 | marihuana.mp. [mp=abstract, heading words, title] |
| 26 | tetrahydrocannabinol.mp. [mp=abstract, heading words, title] |
| 27 | THC.mp. [mp=abstract, heading words, title] |
| 28 | Sativex.mp. [mp=abstract, heading words, title] |
| 29 | dronabinol.mp. [mp=abstract, heading words, title] |
| 30 | 23 or 24 or 25 or 26 or 27 or 28 or 29 |
| 31 | 6 or 30 |
| 32 | 21 and 31 |
| 33 | limit 32 to yr= “2021 -Current” |
| **CENTRAL** | |
| 1 | Cannabidiol/ |
| 2 | CBD.mp. [mp=title, original title, abstract, floating sub-heading word, mesh headings, heading words, keyword] |
| 3 | Cannabidiol.mp. [mp=title, original title, abstract, floating sub-heading word, mesh headings, heading words, keyword] |
| 4 | Epidiolex.mp. [mp=title, original title, abstract, floating sub-heading word, mesh headings, heading words, keyword] |
| 5 | Epidyolex.mp. [mp=title, original title, abstract, floating sub-heading word, mesh headings, heading words, keyword] |
| 6 | hemp.mp. [mp=title, original title, abstract, floating sub-heading word, mesh headings, heading words, keyword] |
| 7 | 1 or 2 or 3 or 4 or 5 or 6 |
| 8 | cannabis/ |
| 9 | cannabinoids/ or dronabinol/ |
| 10 | cannabi*.mp. [mp=title, original title, abstract, floating sub-heading word, mesh headings, heading words, keyword] |
| 11 | marijuana.mp. [mp=title, original title, abstract, floating sub-heading word, mesh headings, heading words, keyword] |
| 12 | marihuana.mp. [mp=title, original title, abstract, floating sub-heading word, mesh headings, heading words, keyword] |
| 13 | tetrahydrocannabinol.mp. [mp=title, original title, abstract, floating sub-heading word, mesh headings, heading words, keyword] |
| 14 | THC.mp. [mp=title, original title, abstract, floating sub-heading word, mesh headings, heading words, keyword] |
| 15 | Sativex.mp. [mp=title, original title, abstract, floating sub-heading word, mesh headings, heading words, keyword] |
| 16 | dronabinol.mp. [mp=title, original title, abstract, floating sub-heading word, mesh headings, heading words, keyword] |
| 17 | 8 or 9 or 10 or 11 or 12 or 13 or 14 or 15 or 16 |
| 18 | 7 or 17 |
| 19 | exp psychomotor performance/ |
| 20 | cognition/ |
| 21 | learning/ |
| 22 | executive function/ |
| 23 | automobile driving/ |
| 24 | reaction time/ |
| 25 | attention/ |
| 26 | memory/ or memory, short-term/ or mental recall/ |
| 27 | cogniti*.mp. [mp=title, original title, abstract, floating sub-heading word, mesh headings, heading words, keyword] |
| 28 | impair*.mp. [mp=title, original title, abstract, floating sub-heading word, mesh headings, heading words, keyword] |
| 29 | psychomotor.mp. [mp=title, original title, abstract, floating sub-heading word, mesh headings, heading words, keyword] |
| 30 | attention.mp. [mp=title, original title, abstract, floating sub-heading word, mesh headings, heading words, keyword] |
| 31 | memory.mp. [mp=title, original title, abstract, floating sub-heading word, mesh headings, heading words, keyword] |
| 32 | reaction time.mp. [mp=title, original title, abstract, floating sub-heading word, mesh headings, heading words, keyword] |
| 33 | coordinat*.mp. [mp=title, original title, abstract, floating sub-heading word, mesh headings, heading words, keyword] |
| 34 | intoxic*.mp. [mp=title, original title, abstract, floating sub-heading word, mesh headings, heading words, keyword] |
| 35 | driving.mp. [mp=title, original title, abstract, floating sub-heading word, mesh headings, heading words, keyword] |
| 36 | task switching.mp. [mp=title, original title, abstract, floating sub-heading word, mesh headings, heading words, keyword] |
| 37 | processing speed.mp. [mp=title, original title, abstract, floating sub-heading word, mesh headings, heading words, keyword] |
| 38 | or/ 19-37 |
| 39 | 18 and 38 |
| 40 | limit 39 to yr= “2022 -Current” |
| **PSYCH INFO** | |
| 1 | CBD.mp. [mp=title, abstract, heading word, table of contents, key concepts, original title, tests & measures, mesh] |
| 2 | Cannabidiol.mp. [mp=title, abstract, heading word, table of contents, key concepts, original title, tests & measures, mesh] |
| 3 | Epidiolex.mp. [mp=title, abstract, heading word, table of contents, key concepts, original title, tests & measures, mesh] |
| 4 | Epidyolex.mp. [mp=title, abstract, heading word, table of contents, key concepts, original title, tests & measures, mesh] |
| 5 | 1 or 2 or 3 or 4 |
| 6 | exp cognitive impairment/ |
| 7 | cognition/ or cognitive processes/ |
| 8 | cognitive ability/ or cognitive flexibility/ or cognitive processing speed/ |
| 9 | cognitive assessment/ or psychological assessment/ or executive functioning measures/ or “memory and learning measures”/ |
| 10 | exp neuropsychological assessment/ |
| 11 | motor coordination/ or motor processes/ or motor control/ or motor skills/ |
| 12 | exp motor performance/ |
| 13 | memory/ or associative memory/ or explicit memory/ or memory consolidation/ or cued recall/ or free recall/ or serial recall/ |
| 14 | exp short term memory/ |
| 15 | spatial navigation/ or spatial perception/ |
| 16 | exp executive function/ |
| 17 | exp attention/ |
| 18 | exp task switching/ |
| 19 | verbal learning/ or paired associate learning/ or “serial anticipation (learning)”/ |
| 20 | discrimination learning/ or stimulus control/ or stimulus discrimination/ |
| 21 | cognitive processing speed. or reaction time/ or response latency/ |
| 22 | driving behavior/ or driving under the influence/ |
| 23 | impair*.mp. [mp=title, abstract, heading word, table of contents, key concepts, original title, tests & measures, mesh] |
| 24 | cogniti*.mp. [mp=title, abstract, heading word, table of contents, key concepts, original title, tests & measures, mesh] |
| 25 | perform*.mp. [mp=title, abstract, heading word, table of contents, key concepts, original title, tests & measures, mesh] |
| 26 | psychomotor.mp. [mp=title, abstract, heading word, table of contents, key concepts, original title, tests & measures, mesh] |
| 27 | attention.mp. [mp=title, abstract, heading word, table of contents, key concepts, original title, tests & measures, mesh] |
| 28 | motor.mp. [mp=title, abstract, heading word, table of contents, key concepts, original title, tests & measures, mesh] |
| 29 | memory.mp. [mp=title, abstract, heading word, table of contents, key concepts, original title, tests & measures, mesh] |
| 30 | reaction time.mp. [mp=title, abstract, heading word, table of contents, key concepts, original title, tests & measures, mesh] |
| 31 | coordinat*.mp. [mp=title, abstract, heading word, table of contents, key concepts, original title, tests & measures, mesh] |
| 32 | concentrat*.mp. [mp=title, abstract, heading word, table of contents, key concepts, original title, tests & measures, mesh] |
| 33 | intoxic*.mp. [mp=title, abstract, heading word, table of contents, key concepts, original title, tests & measures, mesh] |
| 34 | driving.mp. [mp=title, abstract, heading word, table of contents, key concepts, original title, tests & measures, mesh] |
| 35 | task switching.mp. [mp=title, abstract, heading word, table of contents, key concepts, original title, tests & measures, mesh] |
| 36 | processing speed.mp. [mp=title, abstract, heading word, table of contents, key concepts, original title, tests & measures, mesh] |
| 37 | learn*.mp. [mp=title, abstract, heading word, table of contents, key concepts, original title, tests & measures, mesh] |
| 38 | or /6-37 |
| 39 | cannabinoids/ |
| 40 | cannabis/ |
| 41 | marijuana/ |
| 42 | marihuana/ |
| 43 | tetrahydrocannabinol/ |
| 44 | cannabi*.mp. [mp=title, abstract, heading word, table of contents, key concepts, original title, tests & measures, mesh] |
| 45 | marijuana.mp. [mp=title, abstract, heading word, table of contents, key concepts, original title, tests & measures, mesh] |
| 46 | tetrahydrocannabinol.mp. [mp=title, abstract, heading word, table of contents, key concepts, original title, tests & measures, mesh] |
| 47 | THC.mp. [mp=title, abstract, heading word, table of contents, key concepts, original title, tests & measures, mesh] |
| 48 | Sativex.mp. [mp=title, abstract, heading word, table of contents, key concepts, original title, tests & measures, mesh] |
| 49 | dronabinol.mp. [mp=title, abstract, heading word, table of contents, key concepts, original title, tests & measures, mesh] |
| 50 | 39 or 40 or 41 or 42 or 43 or 44 or 45 or 46 or 47 or 48 or 49 |
| 51 | 5 or 50 |
| 52 | 38 and 51 |
| 53 | limit 52 to “0300 clinical trial” |
| 54 | limit 53 to “2022 -Current” |
| **Web of Science** | |
| 1 | ((((((((((((ALL=(Cannabidiol)) OR ALL=(CBD)) OR ALL=(Epidiolex)) OR ALL=(Epidyolex)) OR ALL=(hemp)) OR ALL=(cannabinoids)) OR ALL=(cannabis)) OR ALL=(dronabinol)) OR ALL=(marijuana)) OR ALL=(marihuana)) OR  ALL=(tetrahydrocannabinol)) OR ALL=(THC)) OR ALL=(sativex) |
| 2 | ((((((((((((ALL=(impair*)) OR ALL=(cognit*)) OR ALL=(psychomotor performance)) OR ALL=(attention)) OR ALL=(memory)) OR ALL=(reaction time)) OR ALL=(coordinat*)) OR ALL=(intoxic*)) OR ALL=(driving)) OR ALL=(task switching)) OR ALL=(processing speed)) OR ALL=(executive function)) OR ALL=(learning) |
| 3 | (#1) and #2 and Articles (Document Types) |
| 4 | (TS= clinical trial* OR TS=research design OR TS=comparative stud* OR TS=evaluation stud* OR TS=controlled trial* OR TS=follow-up stud* OR TS=prospective stud* OR TS=random* OR TS=placebo* OR TS=(single blind*) OR TS=(double blind*)) |
| 5 | #5 AND #6 |
| 6 |  |
|  |  |
| **CINAHL** | |
| 1 | (randomized controlled trials OR MH double-blind studies OR MH single-blind studies OR  MH random assignment OR MH pretest-posttest design OR MH cluster sample OR TI  (randomised OR randomized) OR AB (random*) OR TI (trial) OR (MH (sample size) AND  AB (assigned OR allocated OR control)) OR MH (placebos) OR PT (randomized controlled  trial) OR AB (control W5 group) OR MH (crossover design) OR MH (comparative studies)  OR AB (cluster W3 RCT)) NOT ((MH animals+ OR MH animal studies OR TI animal  model*) NOT MH human) |
|  |  |
|  |  |
| **Search Strategy** | |
| S1 | (MH “Cannabidiol”) |
| S2 | TX cannabidiol or cbd |
| S3 | TX epidiolex or epidyolex |
| S4 | TX Hemp |
| S5 | (S1 OR S2 OR S3 OR S4 OR S5) |
| S6 | (MH “Cannabis”) |
| S7 | TX cannabis or marijuana or thc or tetrahydrocannabinol or dronabinol or nabiximols or sativex |
| S8 | (S6 OR S7) |
| S9 | (MH “Memory”) |
| S10 | (MH “executive function”) |
| S11 | (MH “Psychomotor performance”) |
| S12 | (MH “cognition”) |
| S13 | (MH “learning”) |
| S14 | TX intoxication OR TX impairment OR TX memory OR TX attention OR TX ( psychomotor performance or psychomotor activity ) OR TX ( cognition or cognitive function or cognitive performance or cognitive abilities or cognitive ability ) OR TX ( verbal learning or paired associate learning or discrimination learning ) OR TX ( reaction time or response time ) OR TX ( task performance or task switching ) OR TX processing speed OR TX neuropsychological testing |
| S15 | (S9 OR S10 OR S11 OR S12 OR S13 OR S14) |
| S16 | (S5 OR S8) |
| S17 | (S15 AND S16) |
| S18 | (randomized controlled trials OR MH double-blind studies OR MH single-blind studies OR MH random assignment OR MH pretest-posttest design OR MH cluster sample OR TI (randomised OR randomized) OR AB (random*) OR TI (trial) OR (MH (sample size) AND AB (assigned OR allocated OR control)) OR MH (placebos) OR PT (randomized controlled trial) OR AB (control W5 group) OR MH (crossover design) OR MH (comparative studies) OR AB (cluster W3 RCT)) NOT ((MH animals+ OR MH animal studies OR TI animal model*) NOT MH human) |
| S19 | (S17 AND S18) |

1. **eTable 2. PICOS statement**

| **Component** | **Criteria** |
| --- | --- |
| P (Population) | Participants of any age, gender and ethnicity with or without a medical condition |
| I (Intervention) | Acute CBD consumption (any route of administration) |
| C (Comparison) | Placebo controls and THC positive control |
| O (Outcome) | Objective or subjective impairment measures* within 0-8 hours of CBD consumption |
| S (Study type selected) | Clinical or experimental trials, irrespective of design |

*Subjective impairment measures were restricted to those with specific constructs of impairment (e.g., drowsiness, sedation, trouble with memory). Subjective ratings of drug high and intoxication were excluded due to lack of specificity.

1. **eResults**

**Qualitative findings**

At the individual study level, evidence of impairment following acute CBD exposure was only noted in three studies [6–8]. In Solowij (2019), at a vapourized dose of 400 mg purified CBD, infrequent cannabis users self-reported greater drowsiness compared to placebo, but the reverse pattern was observed in more frequent users. In Schoedel (2018), oral Epidiolex doses of 750 mg, 1500 mg, and 4500 mg showed significantly increased subjective drowsiness relative to placebo. However, CBD had significantly less drowsiness compared to the positive controls of alprazolam 2mg, dronabinol 10 mg, and dronabinol 30 mg. Hindocha (2018) observed increased commission errors on the Go/No-Go task compared to placebo with an 800 mg oral synthetic CBD dose, indicating some impairment in executive function. All three studies noted no effect of CBD on other subjective and objective measures of impairment. Arkell (2020) did not report any impairment associated with CBD; however, it was noted that effect sizes for vapourized CBD-dominant cannabis (Δ9-THC < 1% and CBD 9%) may not exclude clinically important impairment.

All other studies reported acute CBD administration, ranging from doses as low as 12.5 mg to as high as 1500 mg, was not associated with impairment in neurocognitive function. This included tests of driving, information processing, working memory, and attention. Further, other studies also assessing subjective drowsiness/sedation and executive function reported null results. In contrast, CBD had improved performance compared to placebo in three trials for tasks assessing sensory discrimination [9], working memory [10], and episodic memory [11].

Nine studies included a Δ9-THC arm for comparison with placebo [7–10,12–16], four of which compared impairment outcomes between Δ9-THC and CBD arms [8,9,14,15]. At oral doses of 600 mg CBD and 10 mg Δ9-THC, Bhattacharyya (2012) reported a significant reduction in response latency (i.e., improved performance) in the visual oddball task for both Δ9-THC and CBD treatment arms compared to placebo, with a trend towards reduced accuracy (i.e., performance impairment) for the Δ9-THC arm only. At these same oral doses, Borgwardt (2008) found no significant differences in Go/No-Go task performance across Δ9-THC, CBD, and placebo treatment arms. However, participants self-reported increased sedation after acute Δ9-THC compared to placebo, but no increase in sedation was reported after CBD [14]. Self-reported sedation/drowsiness, as measured by the VAS, was significantly greater with vapourized Δ9-THC 8 mg than CBD 400 mg, with a tendency for this contrast to be more pronounced in infrequent cannabis users [8]. In van de Donk (2019), the administration of Bedrolite, a CBD-dominant product (18.4 mg CBD + < 1 mg Δ9-THC), was associated with significantly greater alertness compared to both Δ9-THC-dominant products Bedrocan (22.4 mg Δ9-THC + < 1 mg CBD) and Bediol (13.4 mg Δ9-THC + 17.8 mg CBD). Additionally, Bedrolite produced significantly less intense drug high responses and psychoactive effects on internal and external perception compared to both Bedrocan and Bediol [15].

**Quality of evidence**

Of the 20 clinical trials analyzed, three (15%) were deemed to have an overall ‘low risk’ of bias, 16 (76%) were assessed as having ‘some concerns’, and one (5%) was identified as ‘high risk’ of bias. The studies displayed some flaws with respect to control of selection bias and outcome reporting bias. In some cases, minimal information was provided regarding the concealment allocation process or the method of randomization. Other studies did not provide effect sizes or present all data from the analyses described in their methodology.

Egger’s test for funnel plot asymmetry was not statistically significant, consistent with the funnel plot visual (See eFigure 2). Trim and fill methods accordingly showed no change in the effect size estimate. The leave-one-out approach through Jackknife analysis suggested some influential cases. However, variation was minor and centered around the omnibus estimate (range of *g* values = 0.087 to 0.130).

1. **eTable 3. Key characteristics of included trials**

eTable 3. Key characteristics of all included trials.

|  | Trials  (n) | Outcomes  (n) |  | Measure type (n outcomes) |  | Route of administration (n outcomes) | CBD dose (mg)  (Mean, +/ SD) |
| --- | --- | --- | --- | --- | --- | --- | --- |
| All trials | 20 | 427 | Subjective: | 113 | Oral:  Inhaled: | 92  21 | 1115 +/ 1307  77 +/ 114 |
|  |  |  | Objective: | 314 | Oral:  Inhaled: | 272  42 | 1590 +/1546,  39 +/39 |
| Trials eligible for primary quantitative synthesis | 16 | 155 | Subjective: | 33 | Oral:  Inhaled: | 22  11 | 722 +/922  131 +/138 |
|  |  |  | Objective: | 122 | Oral:  Inhaled: | 90  32 | 1101 +/ 1311  47 +/42 |
| Trials ineligible for primary quantitative synthesis | 4 | 7 | Subjective: | 3 | Oral:  Inhaled: | 3  0 | 600 +/ 0 |
|  |  |  | Objective: | 4 | Oral:  Inhaled: | 4  0 | 750 +/ 100 |

1. **eTable 4. Studies by outcome measure included in the quantitative synthesis**

**eTable 4. Studies by outcome measure included in the quantitative synthesis**

| Outcome measure | Study |
| --- | --- |
| Cancellation test | Consroe et al., 1979 |
| Divided attention task (DAT) | Arkell et al., 2020, McCartney et al., 2022, Schoedel et al., 2018, Spindle et al., 2020 |
| Delayed free recall | Hotz et al., 2021 |
| Differential aptitude test | Consroe et al., 1979 |
| Digit span task (DST) | Bloomfield et al., 2020 |
| Driving: on-road | Arkell et al., 2020, McCartney et al., 2022 |
| DRUID test | McCartney et al., 2022 |
| Digit symbol substitution task (DSST) | Arkell et al., 2020, McCartney et al., 2022, Spindle et al., 2020, Schoedel et al., 2018 |
| Finger tap test | Consroe et al., 1979 |
| Fluency test | Morgan et al., 2018 |
| Go/No-Go task | Borgwardt et al., 2008, Hindocha et al., 2018 |
| Hopkins verbal learning test – revised (HVLT-R) | Schoedel et al., 2018 |
| N-back | Bloomfield et al., 2020, Hotz et al., 2021, Hindocha et al., 2018, Morgan et al., 2018 |
| Paced serial addition task (PSAT) | Arkell et al., 2020, McCartney et al., 2022, Spindle et al., 2020 |
| Prose recall | Bloomfield et al., 2020, Morgan et al., 2018, Hindocha et al., 2018 |
| Psychomotor vigilance task (PVT) | McCartney et al., 2022 |
| Time production task | Consroe et al., 1979 |
| Tower of London (TOL) | Arkell et al., 2020 |
| Trailmaking test part a (TMT-A) | Morgan et al., 2018 |
| Trailmaking test part b (TMT-B) | Morgan et al., 2018 |
| Visual analog scale (VAS)/ Visual analog mood scale (VAMS) - sedation/tired/fatigue | Arkell et al., 2020, Arout et al., 2022, Bhattacharyya et al., 2009, Borgwardt et al., 2008 Hotz et al., 2021, McCartney et al., 2022, Schoedel et al., 2018, Solowij et al., 2019, Spindle et al., 2020, van de Donk et al., 2019 |
| Visual analog scale (VAS) - alert/stimulated | Arout et al., 2022, McCartney et al., 2022, Spindle et al., 2020 |
| Visual oddball task (VOT) | Bhattacharyya et al., 2012 |
| Verbal paired associate learning (VPAL) | Bhattacharyya et al., 2009, O’Neill et al., 2021 |

1. **eFigure 1. Risk of Bias assessment**

eFigure 1. Risk of bias assessments. A = crossover trials. B = parallel trials.

1. **eFigure 2. Funnel plot**


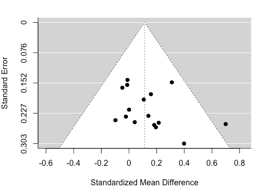


eFigure 2. Funnel plot to assess publication bias.

References:

1. Spindle TR, Cone EJ, Goffi E, Weerts EM, Mitchell JM, Winecker RE, et al. Pharmacodynamic effects of vaporized and oral cannabidiol (CBD) and vaporized CBD-dominant cannabis in infrequent cannabis users. Drug and Alcohol Dependence. 2020;211:107937.

2. Vandrey R, Herrmann ES, Mitchell JM, Bigelow GE, Flegel R, LoDico C, et al. Pharmacokinetic Profile of Oral Cannabis in Humans: Blood and Oral Fluid Disposition and Relation to Pharmacodynamic Outcomes. Journal of Analytical Toxicology. 2017;41:83–99.

3. Zamarripa CA, Vandrey R, Spindle TR. Factors that Impact the Pharmacokinetic and Pharmacodynamic Effects of Cannabis: a Review of Human Laboratory Studies. Curr Addict Rep. 2022;9:608–621.

4. McCartney D, Arkell TR, Irwin C, McGregor IS. Determining the magnitude and duration of acute Δ9-tetrahydrocannabinol (Δ9-THC)-induced driving and cognitive impairment: A systematic and meta-analytic review. Neuroscience & Biobehavioral Reviews. 2021:S0149763421000178.

5. Potvin S, Pelletier J, Grot S, Hébert C, Barr AM, Lecomte T. Cognitive deficits in individuals with methamphetamine use disorder: A meta-analysis. Addictive Behaviors. 2018;80:154–160.

6. Hindocha C, Freeman TP, Grabski M, Crudgington H, Davies AC, Stroud JB, et al. The effects of cannabidiol on impulsivity and memory during abstinence in cigarette dependent smokers. Sci Rep. 2018;8:7568.

7. Schoedel KA, Szeto I, Setnik B, Sellers EM, Levy-Cooperman N, Mills C, et al. Abuse potential assessment of cannabidiol (CBD) in recreational polydrug users: A randomized, double-blind, controlled trial. Epilepsy & Behavior. 2018;88:162–171.

8. Solowij N, Broyd S, Greenwood L, Van Hell H, Martelozzo D, Rueb K, et al. A randomised controlled trial of vaporised Δ9-tetrahydrocannabinol and cannabidiol alone and in combination in frequent and infrequent cannabis users: acute intoxication effects. Eur Arch Psychiatry Clin Neurosci. 2019;269:17–35.

9. Bhattacharyya S, Crippa JA, Allen P, Martin-Santos R, Borgwardt S, Fusar-Poli P, et al. Induction of Psychosis byΔ9-Tetrahydrocannabinol Reflects Modulation of Prefrontal and Striatal Function During Attentional Salience Processing. Archives of General Psychiatry. 2012;69:27–36.

10. Morgan CJA, Freeman TP, Hindocha C, Schafer G, Gardner C, Curran HV. Individual and combined effects of acute delta-9-tetrahydrocannabinol and cannabidiol on psychotomimetic symptoms and memory function. Transl Psychiatry. 2018;8:181.

11. Hotz J, Fehlmann B, Papassotiropoulos A, De Quervain DJf, Schicktanz NS. Cannabidiol enhances verbal episodic memory in healthy young participants: A randomized clinical trial. Journal of Psychiatric Research. 2021;143:327–333.

12. Arkell TR, Vinckenbosch F, Kevin RC, Theunissen EL, McGregor IS, Ramaekers JG. Effect of Cannabidiol and Δ ^9^ -Tetrahydrocannabinol on Driving Performance: A Randomized Clinical Trial. JAMA. 2020;324:2177.

13. Bhattacharyya S, Fusar-Poli P, Borgwardt S, Martin-Santos R, Nosarti C, O’Carroll C, et al. Modulation of Mediotemporal and Ventrostriatal Function in Humans by Δ9-Tetrahydrocannabinol: A Neural Basis for the Effects of Cannabis sativa on Learning and Psychosis. Archives of General Psychiatry. 2009;66:442–451.

14. Borgwardt SJ, Allen P, Bhattacharyya S, Fusar-Poli P, Crippa JA, Seal ML, et al. Neural Basis of Δ-9-Tetrahydrocannabinol and Cannabidiol: Effects During Response Inhibition. Biological Psychiatry. 2008;64:966–973.

15. van de Donk T, Niesters M, Kowal MA, Olofsen E, Dahan A, van Velzen M. An experimental randomized study on the analgesic effects of pharmaceutical-grade cannabis in chronic pain patients with fibromyalgia. Pain. 2019;160:860–869.

16. Winton-Brown TT, Allen P, Bhattacharrya S, Borgwardt SJ, Fusar-Poli P, Crippa JA, et al. Modulation of Auditory and Visual Processing by Delta-9-Tetrahydrocannabinol and Cannabidiol: an fMRI Study. Neuropsychopharmacol. 2011;36:1340–1348.
